# Supplementary material for: Age-related differences in immune dynamics during SARS-CoV-2 infection in rhesus macaques
Source: Life Sci Alliance. 2022 Jan 17;5(4):e202101314. doi: 10.26508/lsa.202101314 (PMC8807873; doi:10.26508/lsa.202101314)
Supplement: Supplementary file 1 [file LSA-2021-01314_TableS1.docx]

**Supplemental Table 1. Clinical signs in rhesus macaques inoculated with SARS-CoV-2.**

| **Animal ID** | **Observed clinical signs** | | | |  |
| --- | --- | --- | --- | --- | --- |
|  | **1-3 dpi** | **4-7 dpi** | **8-14 dpi** | **15-21 dpi** | |
| **RMO1** | Pale appearance, reduced activity, mildly depressed, tachypnea | Pale appearance, reduced activity, mildly depressed, ruffled fur, tachypnea, dyspnea | N/A | N/A | |
| **RMO2** | Tachypnea, dyspnea | Slightly reduced appetite, nosebleed on 4 dpi, dyspnea | N/A | N/A | |
| **RMO3** | Pale appearance, reduced activity, ruffled fur, dyspnea | Pale appearance, reduced activity, slightly ruffled fur, slightly reduced appetite, mildly dehydrated, dyspnea | N/A | N/A | |
| **RMO4** | Ruffled fur, mildly dehydrated | Reduced activity, ruffled fur, severely reduced appetite, mildly dehydrated, dyspnea | N/A | N/A | |
| **RMO5** | Pale appearance, hunched posture, reduced activity, mildly depressed, reduced appetite | Reduced activity, depressed, reduced appetite | Mildly depressed | Mildly depressed, reduced appetite | |
| **RMO6** | Reduced appetite | Reduced appetite | Slightly reduced appetite | Slightly reduced appetite | |
| **RMO7** | Ruffled fur, reduced appetite, tachypnea | Ruffled fur, severely reduced appetite, tachypnea | Ruffled fur, severely reduced appetite, tachypnea | Severely reduced appetite, tachypnea | |
| **RMO8** | Ruffled fur, tachypnea | Ruffled fur, reduced appetite, tachypnea | Ruffled fur, reduced appetite, tachypnea | Tachypnea | |
| **RMY1** | Slightly reduced appetite, tachypnea | Slightly reduced appetite | N/A | N/A | |
| **RMY2** | No signs | Reduced appetite, serous nasal discharge on 6 dpi, dyspnea | N/A | N/A | |
| **RMY3** | Dyspnea | Slightly reduced appetite, dyspnea | N/A | N/A | |
| **RMY4** | Pale appearance, tachypnea, dyspnea | Slightly reduced appetite, dyspnea | N/A | N/A | |
| **RMY5** | Slightly reduced appetite, dyspnea | Dyspnea | No signs | Slightly reduced appetite, recovered by 18 dpi | |
| **RMY6** | Ruffled fur, reduced appetite, tachypnea | Ruffled fur, tachypnea, dyspnea | Dyspnea | Recovered | |
| **RMY7** | Slightly reduced appetite, tachypnea | Slightly reduced appetite, tachypnea | Slightly reduced appetite, tachypnea | Slightly reduced appetite, tachypnea through 17 dpi | |
| **RMY8** | Ruffled fur, reduced appetite | Hunched posture, ruffled fur, tachypnea | Dyspnea | Recovered | |
